# The effect of earthworm extract on promoting skin wound healing

**DOI:** 10.1042/BSR20171366

**Published:** 2018-04-20

**Authors:** Zhen-han Deng, Jian-jian Yin, Wei Luo, Ronak Naveenchandra Kotian, Shan-shan Gao, Zi-qing Yi, Wen-feng Xiao, Wen-ping Li, Yu-sheng Li

**Affiliations:** 1 Department of Orthopaedics, Xiangya Hospital, Central South University, Changsha, Hunan, P.R. China; 2 Department of Orthopaedics, Shenzhen Second People’s Hospital (The First Hospital Affiliated to Shenzhen University), Shenzhen, Guangdong, China; 3 Department of Animal Science, College of Animal Science and Technology, Hunan Agricultural University, Changsha, Hunan, P.R. China; 4 Department of Orthopaedics, Victoria Hospital, Bangalore Medical College and Research Institute, Bangalore, India; 5 Department of Orthopaedic Surgery, University of Texas Health Science Center at Houston, Houston, TX, U.S.A.

**Keywords:** Earthworm extract, Hyp, IL-6, TGF-β, Wound healing

## Abstract

Chronic nonhealing wounds pose a significant challenge to healthcare system because of its tremendous utilization of resources and time to heal. It has a well-deserved reputation for reducing the quality of life for those affected and represent a substantial economic burden to the healthcare system overall. Earthworms are used as a traditional Chinese medicine, and have been applied pharmacologically and clinically since a long time in China. However, there is paucity in data regarding its wound healing effects. Therefore, we investigated the effect of earthworm extract (EE) on skin wound healing process. The obtained data showed that EE has healing effects on local wound of mice. It decreased the wound healing time and reduced the ill-effects of inflammation as determined by macroscopic, histopathologic, hematologic, and immunohistochemistry parameters. The potential mechanism could be accelerated hydroxyproline and transforming growth factor-β secretion—thus increasing the synthesis of collagen, promoting blood capillary, and fibroblast proliferation. It could accelerate the removal of necrotic tissue and foreign bodies by speeding up the generation of interleukin-6, white blood cells, and platelets. It thus enhances immunity, reduces the risk of infection, and promotes wound healing. All in all, the obtained data demonstrated that EE improves quality of healing and could be used as a propitious wound healing agent.

## Introduction

Skin, the largest organ of the body, acts as the first level of defense that shields the host from the external environment and plays a fundamental role in the homeostasis maintenance. Wounds open the door for the entry of foreign materials and organisms into the body [1]. Large area of skin integrity loss could result in severe disability or even life-threatening consequence [2]. The skin wound healing is a dynamic and complex biological process that involves hemostasis, inflammation, granulation, proliferation, production of matrix, and remodeling [3,4]. Poor skin wound healing represents a serious health problem worldwide, frequently associated with high costs and inefficient treatments [5]. In the recent years, great efforts have been made to look for naturally occurring active components that are capable healing skin wounds and other diseases.

Earthworm (Lumbrucis terrestris) belongs to the annelida phylum (in the animalia kingdom) and is a decomposer in the world-wide ecosystem [6]. The segmented earthworm’s body cavity is filled with coelomic fluid, containing 18 amino acids, fatty acids, microelements, lumbritin, lumbrofebrin, terrestrolum brolysin, purine, choline, cholesterin, and vitamins [7]. Extraction and use of biologically active compounds from earthworms have been traditionally practiced by indigenous people throughout the world, more particularly in Asia, including China, India, Myanmar, Korea, and Vietnam [8]. Previous earthworm studies have shown its antiulceral [9], antioxidative [10], hepatoprotective [11], anti-inflammatory [12], antimicrobial, anticancer [13], antiapoptosis [14,15], anticoagulation, and fibrinolytic activities [16]. All of these properties and action modalities may contribute to the acceleration of wound healing process. Hence, we investigated the influence of earthworm extract (EE) on skin wound healing process versus that of Jingwanhong (JWH), an well-established and widely used ointment for external wounds, and secondarily explore the potential mechanism of action of EE in promoting wound healing.

## Materials and methods

### Ethics approval and consent to participate

All animal care, handling, and surgical techniques followed protocols approved by the Animal Care and Use Committee of Central South Univeristy.

### Preparation of earthworm extract (EE)

Earthworms, Ohira the second, were cultured at a breeding center. Briefly, the live earthworms were kept in distilled water at room temperature for one night to clean attached mud. Next day, earthworms were homogenized with phosphate buffer solution (0.02 M pH 7.2), and then centrifuged (5000×***g***, 4°C, 10 min). The supernatant was ultrafiltered in sequence with 100 KD Millipore, 50 KD Millipore, and 30 KD Millipore ultrafilter at 5000×***g*** and 4°C for 10 min. The final ultrafiltered solution was collected and then stored at −20°C for the following experiments.

### Experimental animals

Sixty KunMing mice with equal proportion of male and female (6–8 weeks of age) were purchased from the animal Laboratory Animal Center of Central South University, China. They were housed in a friendly and environmentally controlled conditions with proper room temperature (23°C), humidity (60%), a 12-h light/12-h dark cycle, access to standard rodent food, and water *ad libitum*.

### Mouse wound model and grouping

Excisional punches were created as previous described with several slight modifications [17]. Mice (±20 g) were anesthetized by intraperitoneal injection of 100 mg/kg ketamine and 20 mg/kg xylazine. The dorsum was depilated and sterilized with iodine solution. Two 3 mm full-thickness excisional wounds were created below the level of the panniculus carnosus, one on each flank of animal, using a sterile, disposable, 1 cm-diameter custom-made biopsy punch. Mice were individually caged and received subcutaneous injections of bupivacaine (2 mg/kg body weight) for postoperative pain relief and strepsin for prevention of wound infection.

On the second day after injury, the mice were randomly assigned into three groups of 20 mice (10 male and 10 female mice) each. To the EE treatment group, 0.1 ml of EE was sprayed on the surface of the wound. To the JWH (Jingwanhong, Tianjin Darentang Jingwanhong Pharmaceutical Co., Ltd., Tianjin, China, No. Z12020440) scald ointment treatment group, 0.1 g of JWH cream was sprayed on the surface of the wound. To the control Group, no treatment was applied. Wound management was performed twice a day until it was closed. The wound was considered to be completely closed when the wound area was grossly equal to zero.

### Macroscopic observation

The number and production of the exudates, contraction, the scab, and skin colors of the wound as well as diet and mental status were observed on days 1, 3, 7, 11, and 15 following treatments.

### Wound healing rate determination

The wound area was measured on days 0, 3, 7, 11, and 15 after the trauma by tracing the wound margin and was calculated using an image analysis program (Image J, NIH, MD, U.S.A.). The wound healing rate (WHR) was calculated using the following formula: Wound healing rate = [(initial wound healing area) − (wound healing area on day *N*)]/(initial wound healing area) × 100%. The date of complete wound closure was recorded and the wound healing time (WHT) was calculated accordingly.

### Histopathological observation

Four mice from each group were killed on days 3, 7, 11, and 15. Wounds with the surrounding skin were excised. The specimen was harvested with a 2-mm border of unwounded skin tissue, and then fixed in 10% formalin and later embedded in paraffin. The tissue blocks were then cut into 5 μm sections, transferred to glass slides for hematoxylin and eosin (H&E) staining (HE staining kit, Nanjing Jiancheng Bioengineering Institute, Nanjing, China, batch No. 20120926). Morphological alterations in the skin tissues were examined by light microscopy and documented by photographs.

### Immunohistochemistry (IHC)

Skin wound samples were cut into 4 μm-thick sections perpendicular to the surface of the wound at the middle of each specimen. A VectastainV R VEC PK-6101 Kit (Vector Labs) was used for IHC staining. Sections were incubated with a primary antibody against Ki-67 (Cat# ab15580; Abcam) at room temperature for 1 h, followed by a biotinylated secondary antibody at room temperature for half an hour. The Ki-67 antigen was visualized using the VectorV R NovaREDTM horseradish peroxidase substrate and the VectorV R NovaREDTM Substrate Kit (Vector Labs) followed by Mayer’s hematoxylin nuclear counterstain.

Images were taken using the same microscope and number of Ki-67-positive cells per field was counted at a magnification of 400× (*n* = 5 fields). Nucleus or cytoplasm stained to brown or claybank were positive cells. The positive cell number index (%) = (number of positive cells/number of total cells) × 100%. According to the percentage of positive cells, expression of Ki-67 protein in dermis of the wound was classified into four degrees: negative (−), no positive cell; weak positive (+), positive cells < 10%; middle positive (++), positive cells 10–60%; strong positive (+++), positive cells > 60%.

### Hematology parameters determination

About 200 μl of mouse whole blood was collected (EDTA as *in vitro* anticoagulant) from caudal vena cava at 6 and 12 h, 1, 3, 7, 11 and 15 days after modeling. Routine blood test was immediately performed on a Sysmex XT-2000i automated hematology analyser (sysmex, Japan) for the white blood cells (WBC), platelets (PLT), and neutrophilic granulocyte (GRAN). To separate the serum samples, blood was collected from retro-orbital plexus with 1.5 ml of Eppendorf tube, then centrifuged at 2500 rpm/min at 4°C for 5 min after 6 h of positioning. The serum samples were then stored at −20°C and analyzed within 48 h of collection.

### Determination of hydroxyproline

On days 3, 7, 11, and 15, the determination of hydroxyproline (Hyp) in serum samples was carried out according to the Hyp alkali hydrolysis kit (Nanjing Jiancheng Bioengineering Institute, Nanjing, China, batch No.20121023) [18].

### Enzyme-linked immunosorbent assay (ELISA)

The serum samples for cytokine analysis were homogenized in protease inhibitor cocktail (Calbiochem) as described by Faunce et al. [19]. The concentrations of interleukin 6 (IL-6) and transforming growth factor-β (TGF-β) were assayed by ELISA according to the manufacturer’s recommendations (R&D Systems). Absorbance was measured at 450 nm on an ELISA plate reader (STAT FAX 2100; Awareness Technology, Inc, Palm City, FL).

### Statistical analysis

The values were expressed as means ± the standard deviation (SD). All statistical analyses were performed using the SPSS 16.0 software (Chicago, IL, U.S.A.). Data were analyzed by the independent samples *t*-test compared with control group. A value of ^a^*P*<0.05 was considered statistically significant (^a^*P*<0.05, ^aa^*P*<0.01).

## Results

### Macroscopic observation

**Figure 1:**
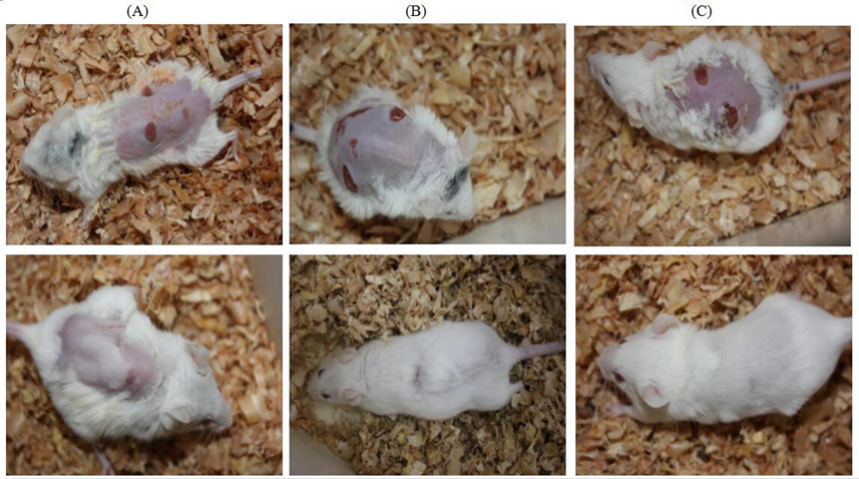
Macroscopic evaluation of wound at the 3rd (upper row) and the 15th day (lower row) in each group (**A**) The control group, (**B**) the JWH group, and (**C**) the EE group.

During modeling and the whole procedure, no pathological reactions like shock, diarrhea, and apastia occurred. Additionally, no mice died of anesthesia, infection, and other complications. On the first day after injury, there was hemorrhage, edema, and exudation around the wound area in all groups. After treatment, the JWH group was the fastest to coagulate, while it took a relative longer time to coagulate in the EE group than the other two groups. Some mice developed the symptoms of slight depression, drowsiness, inactive, fear, vigilance, and several physiological stress related response, but all returned to normal on the second day. On the third day, there was still blood clot on the wound surface in the EE group and the JWH group. The wound surface was fresh with decreased edema and granulation tissue emerged. The wound was dry, slightly red, swollen, and few granulation tissue emerged in the control group. On the seventh day, the wound was wet with much secretion, granulation tissue covered large part of the surface in both the EE group and the JWH group, almost half of the wound was covered with thin layer of epithelial tissue and obvious wound contraction. On the 11th day, the wound began to scab, the wound condition was observed to be the best in EE group with appearance of fur. JWH group was better than the control group. On the 15th day, wound showed complete re-epithelialization with formation of normal basement membrane and fur in all the groups. The EE group was observed to be in better standing when compared to other two groups (Figure 1).

### WHR and WHT

WHR at all time points, WHT, and pairwise comparison between all of the time points of each group are presented in Table 1. The EE group and the JWH group had higher WHR compared with the control group on days 7, 11, and 15 (*P*<0.01). However, no significant difference was observed between the three groups at the third day after modeling (*P*>0.05). When compared with the JWH group, the EE group had higher WHR at 7th (*P*<0.01) and the 11th day (*P*<0.05), while no difference between the two groups was observed at the 15th day (*P*>0.05).

**Table 1:** WHR and WHT of mice in each group (mean ± SD)

Groups	Number	WHR (%)				WHR (d)
		3rd day	7th day	11th day	15th day	
EE	4	13.45 ± 3.02	69.15 ± 6.25^†§^	88.34 ± 2.86^†‡^	100^†^	14.18 ± 0.31^†‡^
JWH	4	12.51 ± 3.34	61.76 ± 2.37^†^	84.99 ± 4.77^†^	100^†^	15.03 ± 0.79^†^
Control	4	10.67 ± 2.36	52.58 ± 4.16	72.65 ± 3.17	97.86 ± 1.78	17.54 ± 0.52

Abbreviations: WHR, wound healing rate; WHT, wound healing time. ^†^*P*<0.01 compared with the control group; ^‡^*P*<0.05 compared with the JWH group; ^§^*P*<0.01 compared with the JWH group.

Both the EE group and the JWH group had shorter WHT than the control group (*P*<0.01), which means shorter wound repair process in these two groups. In addition, there was a significant difference in WHT between the EE group and the JWH group (*P*<0.05), indicating that EE is more effective than JWH in faster healing of the wound.

### Histology

**Figure 2:**
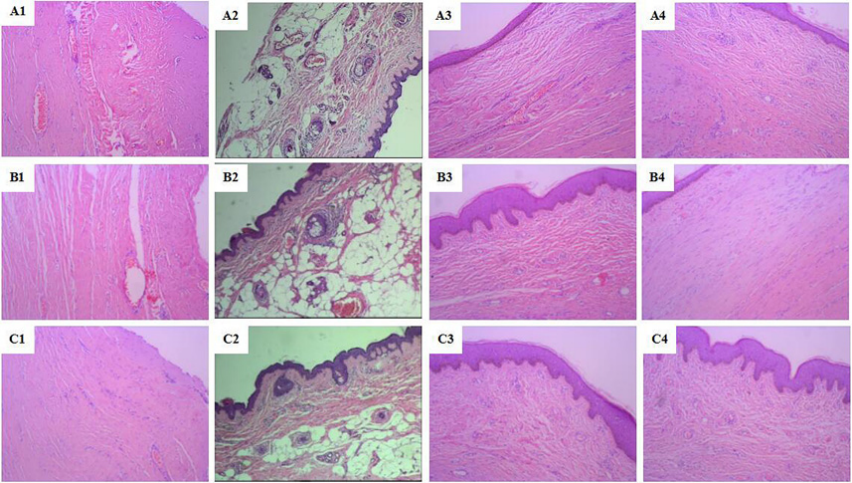
Histological characteristics of wound healing at each time point in each group (H&E staining, ×100) (**A1**), (**B1**), and (**C1**): the third day after modeling; (**A2**), (**B2**), and (**C2**): the 7th day after modeling; (**A3**), (**B3**), and (**C3**): the 11th day after modeling; (**A4**), (**B4**), and (**C4**): the 15th day after modeling; (A1–A4): the control group; (B1–B4): the JWH group; (C1–C4): the EE group.

On the third day after injury, H&E staining showed no epidermis formation in the control group, lots of necrotic tissues, scab, dermal connective tissue tightly combined with large amount of inflammatory cells infiltration, loose intercellular matrix and no obvious signs of new capillaries formation and fibroblast proliferation. In the JWH group, no epidermis formation, clear bound between scab and dermis, inflammatory cells infiltration in connective tissue. No epidermis formation in the EE group, more inflammatory cells infiltration in subcutaneous tissue than the JWH group, intercellular matrix was edematous and loose, few minute vessel expansion, new capillaries formation, and fibroblast proliferation (Figure 2A1,B1,C1).

On the 7th day in the control group, more large dark and radical shaped fibroblast appeared, but the majority was round or oval shaped with light stained cytoplasm and loose intercellular matrix. In the JWH group, there was less inflammatory cells, fibroblasts diffusion but regularly arranged and few capillaries formation. In the EE group, the number of inflammatory cells decreased, large amount of regularly arranged, asteroid or fusiform shaped fibroblasts with big cell body, abundant cytoplasm, much synapses, and the enlarged and oval shaped nucleus were deeper stained, more capillaries formation and few granulation tissue appeared (Figure 2A2,B2,C2).

On the 11th day in the control group, the wound surface was rough, covered by a thin layer of granulation tissue with few loose collagenous fibers. In the JWH group, the wound was covered by a thick layer of epithelial tissue with several connective tissue, radial fibroblasts, and more dense collagenous fibers. The layer of epithelial tissue in the EE group was thicker compared with the JWH group, the wound was fixed by organized and proliferated granulation tissue, part of which turned into connective tissue; large amount of dense mature collagen fibers regularly arranged and more capillaries formation (Figure 2A3,B3,C3).

On the 15th day in the control group, inflammatory cells almost disappeared; capillary number decreased with wider diameter; more larger size, dark nuclear stained fibroblasts appeared, disorderly arranged in bundles. In the JWH group, large amount of collagen fibers filled the intercellular matrix; fibroblasts closely arranged, expanded capillaries, many fat cells appeared in the subcutaneous tissue. In the EE group, large numbers of fusiform shaped mature fibroblasts regularly arranged in bundles; many collagen fibers filled the intercellular substance; even extracellular matrix, more fat cells in subcutaneous tissue and hair follicle formation (Figure 2A4,B4,C4).

### Hematology parameters

There were more PLT in the EE group and the JWH group compared with the control group at 3, 7, and 11 days (*P*<0.05 or *P*<0.01, Table 2). The number of PLT on third day was higher compared with the 15th day, suggesting PLT number raised at early stage of injury, then reached its peak on the 7th day with significant difference between the EE group and the JWH group at that time point (*P*<0.05). The number of PLT decreased on the 11th day without any significant difference between the three groups until the 15th day (*P*>0.05).

**Table 2:** PLT, WBC, and GRAN numbers of mice in each group (10^9^/l, mean ± SD)

	Group	3rd day	7th day	11th day	15th day
PLT	EE	462 ± 11.23*^†^*	504 ± 19.33^†^*^‡^*	442 ± 4.66*^†^*	363 ± 21.01
	JWH	456 ± 5.66*^†^*	471 ± 7.76*^†^*	431 ± 5.83*	371 ± 30.08
	Control	413 ± 2.53	432 ± 6.35	415 ± 17.76	372 ± 25.33
WBC	EE	13.52 ± 0.65^†§^	13.07 ± 0.18*^†^*	11.22 ± 0.32	8.74 ± 0.14
	JWH	11.73 ± 0.89*^†^*	12.90 ± 0.27*^†^*	10.84 ± 0.44	8.49 ± 0.52
	Control	9.58 ± 0.23	10.40 ± 0.59	11.03 ± 0.36	8.24 ± 0.33
	**Group**	**6th h**	**12th h**	**24th h**	**72th h**
GRAN	EE	213 ± 10.22*^†^*	325 ± 20.38^†^§^^	235 ± 12.45^†^*^‡^*	9 ± 2.24
	JWH	196 ± 9.67*^†^*	267 ± 15.76*^†^*	179 ± 8.85*^†^*	8 ± 1.67
	Control	48 ± 3.23	62 ± 9.31	54 ± 5.33	8 ± 1.13

Abbreviations: GRAN, neutrophilic granulocyte; PLT, platelet; WBC, white blood cell. *P<0.05 compared with the control group; *^†^P*<0.01 compared with the control group; *^‡^P*<0.05 compared with the JWH group; *^§^P*<0.01 compared with the JWH group.

There was more WBC in the EE group and the JWH group compared with the control group in the initial 7 days (*P*<0.01, Table 2). The number of WBC stayed at a higher level and reached the peak on the third day with significant difference between the EE group and the JWH group (*P*<0.05). There was no difference between the three groups from the 11th day to the 15th day. WBC count in the control group raised in the initial 11 days and decreased to normal level since then.

The three groups showed similar trend of GRAN number changes. During the post-trauma 6 h, GRAN number elevated swiftly, reached its peak at the 12th h and then started to fall after 24 h. The number of GRAN was significantly higher in the EE group and the JWH group than the control group during the first 24 h (*P*<0.01), and the EE group was higher than the JWH group from the 12 to 24 h (*P*<0.05 or *P*<0.01, Table 2), indicating that EE significantly activated GRAN gathering and differentiation in blood after trauma and was more effective than JWH. There was no difference between the three groups at the 72th hour (*P*>0.05).

### Immunohistochemistry

**Figure 3:**
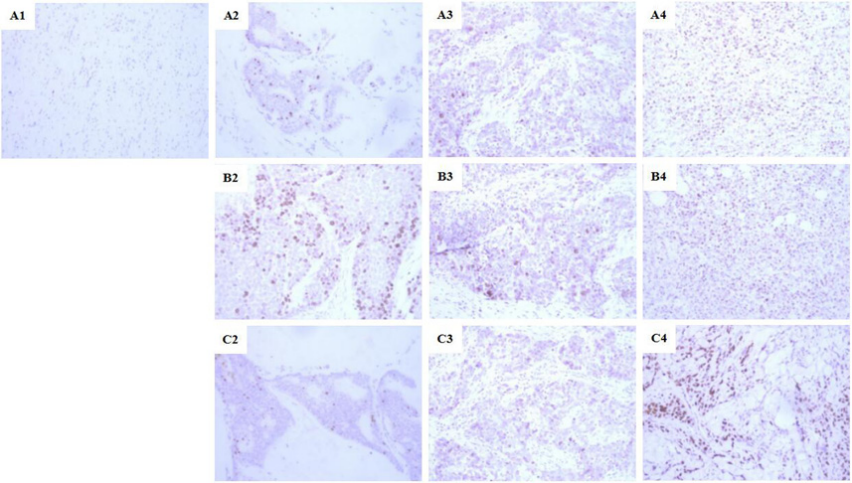
Ki67 protein expression at different grades in each group (×400) (**A1**): negative expression (−); (**A2**), (**B2**), and (**C2**): weak positive expression (+); (**A3**), (**B3**), and (**C3**): middle positive expression (++); (**A4**), (**B4**), and (**C4**): strong positive expression (+++); (A1–A4): the control group; (B2–B4): the JWH group; (C2–C4): the EE group.

Ki-67 protein in fibroblasts is a kind of antigen solely expressed in proliferate nuclears. The expression of Ki-67 differed between the three groups (*P*<0.05, Table 3). On the third day after injury, no Ki-67 expression was detected in the control group. There were two cases of weak positive and one case of positive in the EE group, while one case each in the JWH group. No strong positive case in both the groups, but a higher positive expression rate in the EE group compared with the JWH group. At 7, 11, and 15 days, the rate of weak and positive cell expression raised in the control group, but still lower than the other two groups. The EE group had higher positive cells rate than the JWH group in all grades, indicating that EE exerted an obvious accelerating influence on proliferation and differentiation of fibroblasts during the process of wound healing in mice, which was more effective than JWH (Figure 3).

**Table 3:** Expression of Ki-67 protein in dermis of the wound in each group

Groups	Days	Numbers	Expression of Ki-67 protein			
			−	+	++	+++
EE	3	4	1	2	1	0
	7	4	0	1	3	0
	11	4	0	1	1	2
	15	4	0	0	1	3
JWH	3	4	0	1	1	2
	7	4	0	0	1	3
	11	4	0	1	1	2
	15	4	1	0	1	2
Control	3	4	4	0	0	0
	7	4	2	2	0	0
	11	4	0	2	2	0
	15	4	0	2	1	1

### Hyp, IL-6, and TGF-β concentration in serum

The content of Hyp in all the groups presented the same trend of change, raised during the early stage then decreased in later periods (Table 4). There was no difference in Hyp content among the three groups in the first week (*P*>0.05). On the 11th and 15th day, both EE group and JWH group had higher Hyp content compared with the control group (*P*<0.05 or *P*<0.01), and the content of Hyp was higher in EE group than the JWH group (*P*<0.05).

**Table 4:** The content of Hyp (μg/ml), IL-6 (pg/ml), and TGF-β (pg/ml) in serum in each group (mean ± SD)

	Group	3rd day	7th day	11th day	15th day
Hyp	EE	19.23 ± 1.03	22.01 ± 1.36	27.24 ± 1.87^†‡^	26.64 ± 2.25^†‡^
	JWH	18.89 ± 1.27	21.76 ± 2.24	25.32 ± 2.12^†^	24.82 ± 2.18*
	Control	18.35 ± 1.19	21.34 ± 1.24	21.86 ± 2.49	22.67 ± 3.16
IL-6	EE	253.71 ± 30.48^†§^	456.37 ± 26.33^†§^	193.67 ± 15.58^†^	62.36 ± 16.36
	JWH	164.33 ± 7.38*	225.54 ± 18.96^†^	201.35 ± 5.33^†^	67.83 ± 5.35
	Control	135.60 ± 14.37	161.34 ± 22.56	150.34 ± 8.39	63.41 ± 3.98
TGF-β	EE	215.33 ± 17.88^†^	208.02 ± 21.45^†‡^	191.63 ± 14.76^†‡^	138.24 ± 6.35
	JWH	203.11 ± 34.72*	187.04 ± 19.61^†^	166.24 ± 11.72*	136.23 ± 3.45
	Control	168.07 ± 9.82	150.76 ± 6.39	141.13 ± 10.33	135.77 ± 4.65

Abbreviations: Hyp, hydroxyproline; TGF-β, transforming growth factor-β; *P<0.05 compared with the control group; ^†^*P*<0.01 compared with the control group; ^‡^*P*<0.05 compared with the JWH group; ^§^*P*<0.01 compared with the JWH group.

The expression of IL-6 in serum of the EE group and the JWH group was higher compared with the control group on the 3rd, 7th, and 11th day (*P*<0.05 or *P*<0.01, Table 4). The EE group had significantly higher IL-6 expression than the JWH group on the 3rd and 7th day (*P*<0.01), indicating that EE had an advantage over JWH in promoting IL-6 secretion at an early stage of inflammation. No difference of IL-6 expression was observed among the three groups on the 15th day (*P*>0.05). In the EE group, the IL-6 expression increased swiftly after modeling and reached its peak on the 7th day, and then decreased. The JWH group and the control group showed a mild increase and then decreased after the 7th day.

The expression level of TGF-β raised during the first 3 days after injury and then declined slowly in all the three groups, indicating that the serum TGF-β contributed mainly at an early stage of trauma (Table 4). The EE group and the JWH group had higher TGF-β expression compared with the control group on the 3rd, 7th, and 11th day (*P*<0.05 or *P*<0.01). The TGF-β expression in the EE group was higher than the JWH group on the 7th and 11th day (*P*<0.05). The TGF-β expression decreased to a normal level in all the three groups without any significant difference between one another (*P*>0.05).

## Discussion

Wound healing is a complex process involving different phases of inflammation, new tissue formation, and remodeling. WHR and WHT are the important indicators to evaluate wound healing [20]. In the present study, mice skin wound model with 1 cm diameter of full layer skin defect was treated with different modalities to evaluate the treatment effect. We found an obvious effect of EE in promoting wound healing in our series of experiments. Most notably, it decreased WHT, reduced the injury of inflammation, and improved the quality of healing.

WBCs, also called leukocytes, are the cells of the immune system that are involved in protecting the body against both infectious disease and foreign invaders [21]. GRAN is a type of phagocyte normally found in the bloodstream. When the body is attacked by bacteria or virus, GRAN is one of the first-responders of inflammatory cells to migrate toward the site of inflammation in a process called chemotaxis and releases chemotactic chemicals to kill and swallow the pathogen [22]. The main physiological functions of PLT include achieving hemostasis, clotting of blood, repairing endothelial cells of blood vessels, and maintaining the integrity of blood vessel walls [23]. In the present study, more WBC, GRAN, and PLT cells were found in the EE and the JWH group compared with the control group, indicating that EE can efficiently promote the proliferation of these blood cells. The number of these cells declined at the later stage of wound healing in all the groups, suggesting that EE could quickly respond to inflammatory changes at an early stage of wound formation, thus accelerating wound clotting and wiping out necrotic tissues by promoting WBC and GRAN proliferation. EE also promoted the repair of injured endothelial cells, which plays an important role in hyperplasia and providing nutrition to the new granulation tissue, by promoting PLT proliferation. On macroscopic observation, we found fresher granulation tissue in the EE group, indicating that EE directly affected the local blood supply of the wound. Large amount of expanded capillaries were observed in the EE group through microscope, which is beneficial to increase local blood capillary permeability, enhance blood circulation and nutrition transportation, induce cells enter damaged tissue, activate the secretion of various cytokines in order to increase the capillary generation, promote deposition of collagen fibers and fibroblasts activation, promote wound fiber effusion, promote granulation tissue and epidermis cover, and repair the wound surface. Above observations suggest that EE effectively decreased the WHT; increased the WHR by accelerating WBC, GRAN, and PLT proliferation; promoted angiogenesis; improved local microcirculation of the wound; accelerated the rate of metabolism of wound tissue, and reduced the injury of inflammation.

Fibroblast plays important roles in granulation formation, wound contraction, matrix synthesis, wound repair, scar formation, and scar less repair by means of growth factors modulation [24]. Collagen fiber is the main structural protein in the extracellular space in various connective tissues, making up approximately 25–35% of the whole-body protein content [25]. Wound healing involves in growth of capillaries, fibroblasts, and collagen. This forms granulation tissue [26]. In the present study, there were more fibroblasts and capillaries in the EE group compared with the control group at 3, 7, and 11 days, indicating that EE promoted the proliferative ability and metabolic activity of cells in the granulation tissue which contributes to wound contraction, matrix synthesis, and wound repair.

Collagen can benefit all stages of wound healing and the content of which can be reflected by Hyp, one of its special degradation product. Hyp is a major component of the protein collagen with a relative stable content, comprising roughly 13.5% of mammalian collagen [27]. Determining the change of content of Hyp can reflect the amount of collagen indirectly. The generation of collagen implies the beginning of wound repair and it also plays a vital role in scar formation [28]. The content of Hyp was generally higher in the EE and the JWH group compared with the control group at the same time point. We found low level of Hyp expression during the first three post-trauma days, then raised to the peak and declined to normal level, indicating that few collagens were generated in the early stage of wound formation, but proliferated quickly during the later stage and promoted wound healing. We inferred that EE accelerated the formation of collagen and extracellular matrix (ECM), advanced the stage of granulation tissue hyperplasia and decreased the WHT. There is paucity in the data regarding the active component of EE which is responsible for above observations and the mechanism remains unknown. We hypothesize that EE activates fibroblasts in the wound tissue, stimulating fiber cells maturity as well as promoting synthesis of collagen from the fibroblasts.

Cytokines have been proved to act as the core of regulation in the process of wound healing. It could improve the body’s immunity by promoting chemotaxis of inflammatory cells, regulating proliferation of vascular endothelial cells and fibroblasts, promoting the formation of ECM in order to regulate the process of wound healing [29,30]. The IL-6 and TGF-β are two of the most important cytokines that regulate wound healing [31]. Low or absent cytokines expression during the wound remodeling will adversely affect the healing process. In contrast, overexpression will result in scar formation. IL-6 is produced by epidermal keratinocytes, dermal fibroblasts, endothelial cells, macrophages and the activated T cells and B cells, and it induces other cells to produce protein and accelerate activation and aggregation of the neutrophilic granulocyte [32]. IL-6 is proinflammatory, and increases adhesion of neutrophils to dermal fibroblasts [33]. IL-6 might also assist wound healing indirectly by modulation of growth factors or their receptors. IL-6-deficient transgenic mice (IL-6 KO) displayed significantly delayed cutaneous wound healing compared with wild-type control animals, while application of IL-6 in an *in vitro* corneal wound healing model, IL-6 effectively increased the WHR [34,35]. TGF-β is a multifunctional growth factor that exerts pleiotropic effects on wound healing by regulating cell proliferation and migration, differentiation, ECM production, and immune modulation [36]. During the acute response to injury, large amount of TGF-β is released by PLT, it being its main storage site. This immediate release after injury plays a critical part in macrophage and fibroblast chemotaxis to the wound [37]. TGF-β released by T cells has been shown to engender collagen synthesis and angiogenesis [38]. It has also been shown to stimulate fibroblasts to contract collagen gels, a finding that suggests a role of this cytokine in wound contraction [39]. TGF-β also facilitates re-epithelialization largely by enhancing migration of keratinocytes after the ligand binds to specific cell surface receptors [40]. In addition, the effect of blocking TGF-β by the application of antibodies to adult cutaneous wounds was found to markedly reduce scarring [41]. Conversely, acceleration of wound healing by TGF-β has also been demonstrated in studies of healing-impaired models [42,43]. The present study proved that EE accelerated the secretion of IL-6 and TGF-β, and thus regulated the inflammatory process of the wound.

Our study was not without any limitations. We have not yet isolated the active molecular substance of EE which is responsible for the modulation and signaling pathway, and future investigations are needed to unravel it. Grossly, we did not observe any adverse effects of EE. As it a natural extract and it has been used by people since ages, we assumed it might not have or have negligible adverse effects. Future studies should ascertain this point.

In conclusion, there was an obvious pro-wound healing effects of EE on local wound of mice. It decreased wound healing time, reduced the injury caused by inflammation, and improved the quality of healing. The potential mechanisms are probably by accelerated Hyp and TGF-β secretion, increased synthesis of collagen, blood capillary, and fibroblast proliferation. Additionally, it accelerates the removal of necrotic tissue and foreign body by speeding up the generation of IL-6, WBC, PLT, and Gran. Thus enhances immunity, relieves the inflammatory reaction of mechanical injury, reduces the risk of infection, and finally promotes wound healing.

## Abbreviations

ECM: extracellular matrix
EE: earthworm extract
EGF: epidermal growth factor
ELISA: enzyme-linked immunosorbent assay
FGF: fibroblast growth factor
GRAN: neutrophilic granulocyte
HE: hematoxylin and eosin
Hyp: hydroxyproline
IHC: immunohistochemistry
IL-6: interleukin 6
JWH: Jingwanhong
PLT: platelet
SD: standard deviation
TGF-β: transforming growth factor-β
WBC: white blood cell
WHR: wound healing rate
WHT: wound healing time
